# Helpless Priming Sends CD8^+^ T Cells on the Road to Exhaustion

**DOI:** 10.3389/fimmu.2020.592569

**Published:** 2020-10-06

**Authors:** Julia Busselaar, Sun Tian, Hans van Eenennaam, Jannie Borst

**Affiliations:** ^1^Department of Immunology and Oncode Institute, Leiden University Medical Center, Leiden, Netherlands; ^2^Aduro Biotech Europe BV, Oss, Netherlands; ^3^AIMM Therapeutics BV, Amsterdam, Netherlands

**Keywords:** CD8^+^ T cell, CD4^+^ T cell, exhaustion, dysfunction, cancer, infection

## Abstract

Persistent antigen exposure in chronic infection and cancer has been proposed to lead to cytotoxic T lymphocyte (CTL) “exhaustion”, i.e., loss of effector function and disease control. Recent work identifies a population of poorly differentiated TCF-1^+^PD-1^+^ CD8^+^ T cells as precursors of the terminally exhausted CTL pool. These “predysfunctional” CTLs are suggested to respond to PD-1 targeted therapy by giving rise to a pool of functional CTLs. Supported by gene expression analyses, we present a model in which lack of CD4^+^ T cell help during CD8^+^ T cell priming results in the formation of predysfunctional CTLs. Our model implies that predysfunctional CTLs are formed during priming and that the remedy for CTL dysfunction is to provide “help” signals for generation of optimal CTL effectors. We substantiate that this may be achieved by engaging CD4^+^ T cells in new CD8^+^ T cell priming, or by combined PD-1 blocking and CD27 agonism with available immunotherapeutic antibodies.

## Introduction

In chronic infection and cancer, CD8^+^ T cells upregulate coinhibitory receptors and display impaired proliferative and cytotoxic capacities, a phenomenon described as “T cell exhaustion”. T cell exhaustion is considered a crucial factor in limiting clinical responses to immunotherapy, but this T cell state is not well understood. Some experts do not envision functions for exhausted T cells, while others surmise a role in host protection (1). Recent data illuminate how exhausted CD8^+^ T cells are formed. The original model proposed that exhausted CD8^+^ T cells develop from effector T cells as a result of chronic stimulation *via* their T cell antigen receptor (TCR) (2). However, new transcriptomic analyses, that include TCR-based lineage tracing, argue that exhausted CD8^+^ T cells are not derived from functional effector cells. Rather, CD8^+^ T cells can attain a “predysfunctional” state early after infection or tumorigenesis that may progress into a terminally exhausted state. It is considered that predysfunctional cells may also be “reinvigorated” to become CTL effectors. Blockade of the PD-1/PD-L1 coinhibitory axis may lead to such reinvigoration. Knowledge about the exact molecular and cellular mechanisms underlying CD8^+^ T cell predysfunction, exhaustion and reinvigoration are clinically relevant in chronic infection and cancer, and likely also in auto-immune and inflammatory diseases.

Here, we first discuss the recent literature on CD8^+^ T cell predysfunction and exhaustion in a key mouse model of chronic virus infection. This work has recently led to the concept that predysfunction and exhaustion represent aspects of a CD8^+^ T cell differentiation pathway, distinct from effector and memory differentiation. By connecting studies on infection and cancer, we integrate supporting arguments for this concept. We synthesize these recent insights into a model of progressive fate commitment of primed CD8^+^ T cells. Supported by gene expression analyses, we introduce the novel perspective that the predysfunctional differentiation state results from CD8^+^ T cell priming in the absence of CD4^+^ T cell help. This viewpoint implies that reinvigoration of predysfunctional CD8^+^ T cells may be achieved by addition of “help” signals. We rationalize that PD-1 targeted checkpoint blockade may lead to delivery of help signals and may be supported by engagement of specific T cell costimulatory receptors.

## Methods

### No Help CD8^+^ T Cell Gene Expression Signature

RNAseq fastq files of samples of helped CD8^+^ T cells (n = 3) and samples of non-helped CD8^+^ T cells (n = 3) were retrieved from GEO database (GSE89665) (3). FASTQ files were aligned to the mouse genome mm10 (GRCm38.77) using HISAT2 v2.1.0 (4), and number of reads was assigned to genes by using featureCounts v1.6.1 (5). Reads mapped to genes were normalized and differentially expressed gene analysis between non-helped CD8^+^ T cells and helped CD8^+^ T cell was performed using edgeR package in R Bioconductor (6). The false discovery rate (FDR) < 0.01 was used as the criteria to select statistically differentially expressed gene lists. In total, a list of 1,331 genes were found differentially expressed between non-helped condition and helped conditions (FDR < 0.01), which represents the No Help signature.

### Calculation of No Help Score in Published CD8 T Cell Expression Signatures

RNAseq fastq files were retrieved from GEO database (GSE99531, GSE122713) (7, 8). FASTQ files were aligned to the mouse genome mm10 using HISAT2 v2.1.0 (4), and number of reads was assigned to genes by using featureCounts v1.6.1 (5). Genes with all zero counts were removed. The raw counts were normalized by count per million (CPM) methods (6). For each sample, a “No Help score” was determined by the nearest centroid method on the 1331 genes from the No Help signature. In short, the No Help score was calculated as the difference of Pearson correlations in normalized read counts between a given population and No Help or Help vaccination settings. A higher No Help score indicates greater transcriptional similarity to helpless CD8^+^ T cells.

### Gene Set Enrichment Analysis

RNAseq files of helped or non-helped CD8 T cells, aligned to the mouse genome mm10, were imported into Qlucore Omics Explorer. Genes with less than 5 reads in at least one of the samples were discarded. Mapping quality threshold was set to 10. TNM normalization method was applied. Gene Set Enrichment Analysis was performed using published gene sets of the top 200 up- and downregulated genes from *Tcf7*-GFP^+^ versus *Tcf7*-GFP^−^ P14 cells in chronic LCMV infection (9) or B16-gp33 tumor model (10).

### Statistical Analysis

Data was analyzed with GraphPad Prism software using unpaired two-tailed Student’s t-test, or repeated measures one-way ANOVA with Tukey’s multiple comparison test. A P value < 0.05 was considered statistically significant; *P < 0.05, **P < 0.01, and ***P < 0.001.

### Illustrations

**Figure 1:**
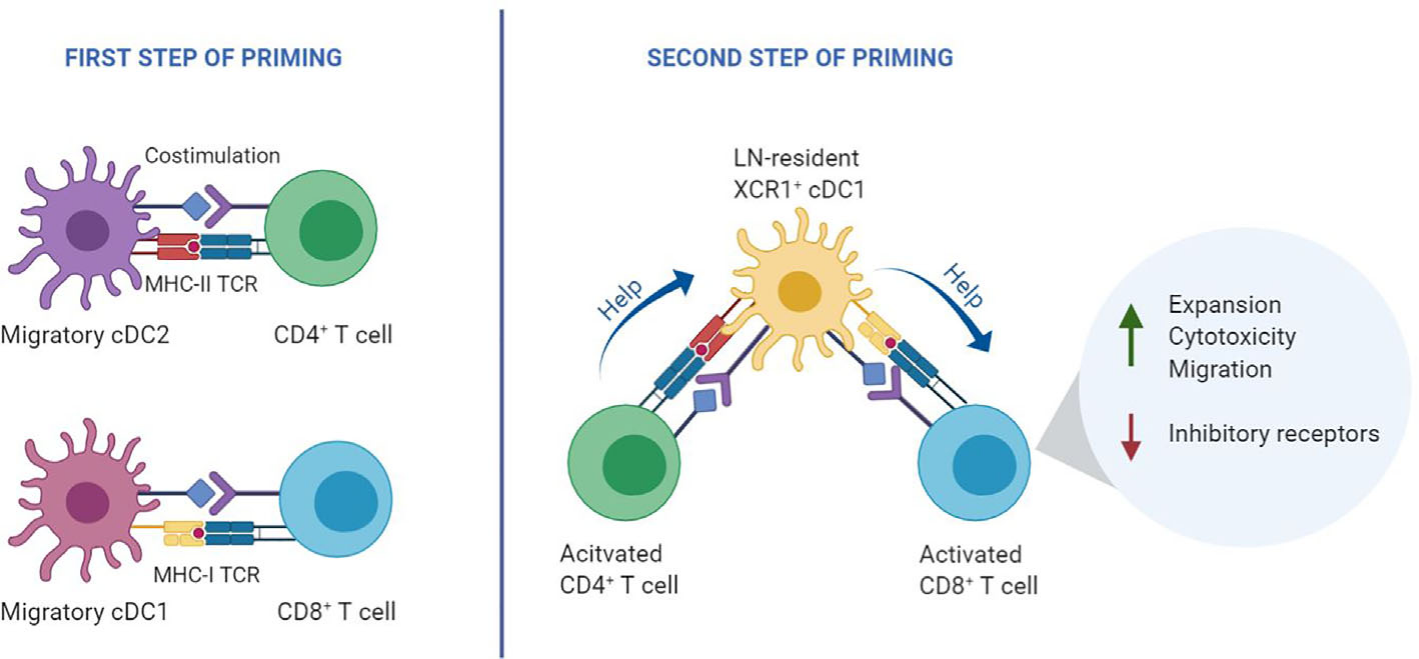
Two-step priming model. During the first step of T cell priming (left), CD8^+^ T cells and CD4^+^ T cells are initially activated independently by different DC subtypes that present antigen on MHC class I and class II, respectively. In the second step of priming (right), recently activated CD4^+^ and CD8^+^ T cells interact with the same lymph node-resident cDC1 co-expressing MHC class I and MHC class II epitopes. Helped CD8^+^ T cells undergo optimal priming by signaling *via* various costimulatory and cytokine signals that emerge from the helped cDC1, resulting in an optimal CTL effector program (11).

**Figure 2:**
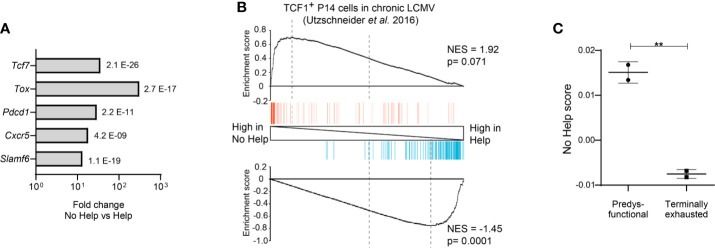
Predysfunctional TCF-1^+^ CD8^+^ T cells in a chronic LCMV infection model display a gene expression signature characteristic of helpless antigen-specific CD8^+^ T cells in a vaccination model. The transcriptional “No Help” signature was determined by differential gene expression (False discovery rate (FDR) < 0.01) of antigen-specific CTLs raised in No Help versus Help vaccination settings (GEO database GSE89665) (3). **(A)** Differential expression of selected genes characteristic of predysfunctional TCF-1^+^CD8^+^ T cells (**Table 1**) in No Help versus Help settings. FDR is depicted per gene. **(B)** GSEA of the top 200 upregulated (red)- or downregulated (blue) genes from TCF1^+^ versus TCF1^−^ virus-specific CD8^+^ T cells in chronic LCMV infection (9) within the gene expression profiles of CD8^+^ T cells from the No Help versus Help vaccination settings. NES, normalized enrichment score. **(C)** No Help score in predysfunctional TCF-1^+^TIM3^−^ and terminally exhausted TCF-1^−^TIM3^+^ CD8^+^ T cells from a setting of chronic LCMV infection (GEO database GSE122713) (7). The No Help score was calculated as the difference of correlations in gene expression between a given population and No Help or Help vaccination settings. A higher No-Help score indicates greater transcriptional similarity to helpless CD8^+^ T cells. **P < 0.01 by Student’s t-test.

**Figure 3:**
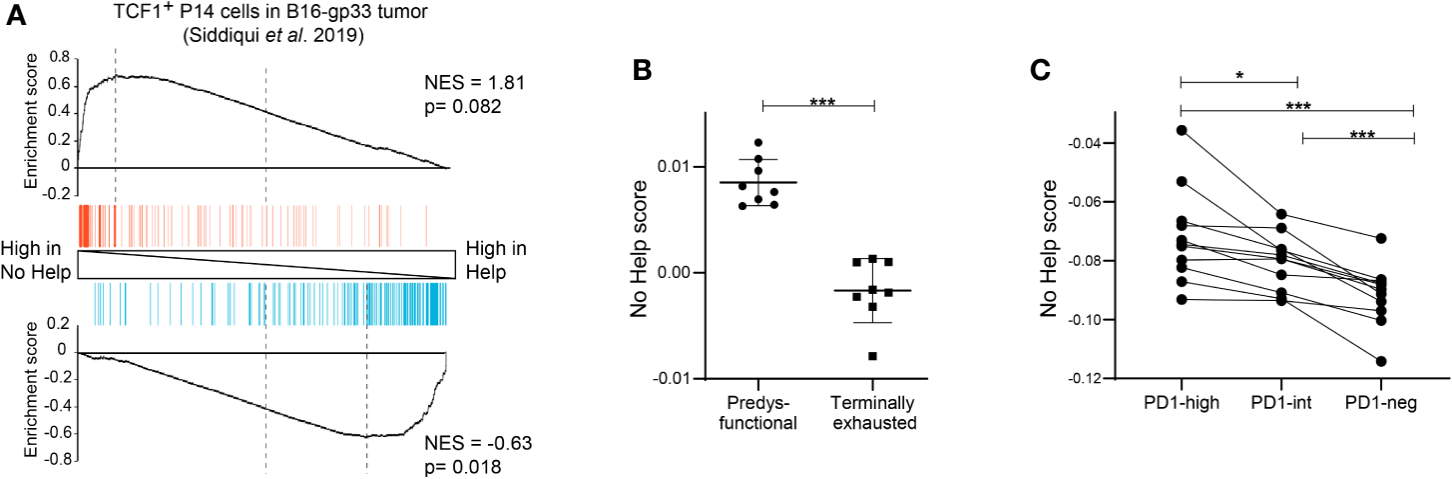
Predysfunctional TCF-1^+^ CD8^+^ T cells in human cancer display a gene expression signature characteristic of helpless antigen-specific CD8^+^ T cells in a mouse vaccination model. **(A)** GSEA showing enrichment of the top 200 upregulated (red) or downregulated (blue) genes in gp33-specific TCF-1^+^ CD8^+^ T cells in a murine B16-gp33 tumor model (10) within the gene expression profiles of vaccine antigen-specific CD8^+^ T cells in No Help versus Help settings (3). **(B)** No Help scores, defined in our vaccination model, determined in the transcriptomes of predysfunctional TCF-1^+^TIM3^−^ and terminally exhausted TCF-1^−^TIM3^+^ tumor antigen-specific CD8^+^ T cells from a murine B16-OVA tumor model (GEO database GSE122713) (7). **(C)** No Help score defined as in **(B)**, determined in the transcriptome of patient-matched PD-1-high, PD-1-intermediate, and PD-1-negative CD8^+^ TILs in human melanoma (GEO database GSE99531) (8). *P < 0.05, ***P < 0.001 by Student’s t-test **(B)** or one-way ANOVA **(C)**.

**Figure 4:**
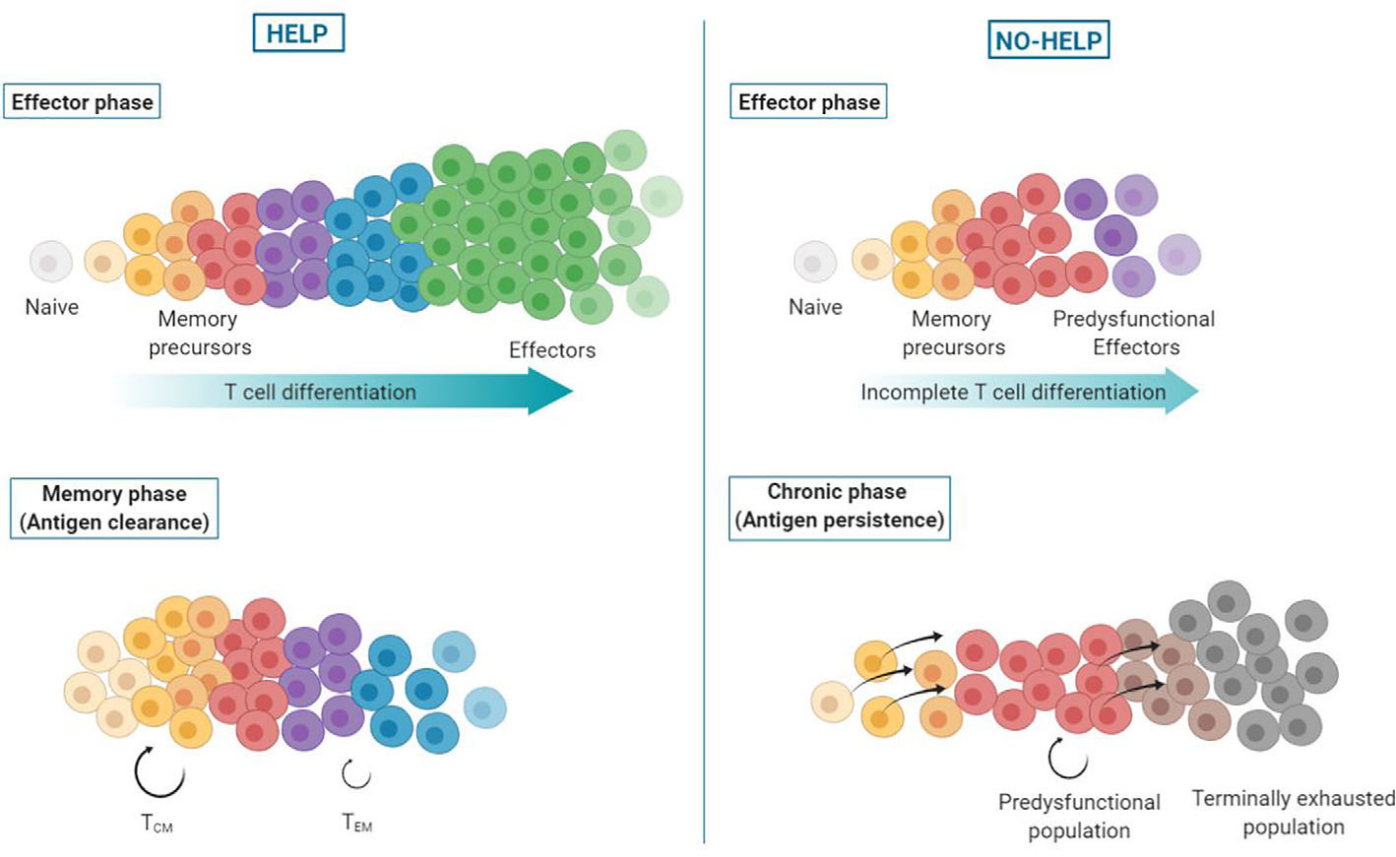
Helpless dysfunction model. Upon priming of CD8^+^ T cells, a differentiation spectrum is formed, ranging from uncommitted memory precursors to terminally differentiated effector cells. In presence of CD4^+^ T cell help signals (left), the antigen-specific CD8^+^ T cell population attains higher differentiation states, with the majority of cells becoming terminally differentiated, short-lived effector CTLs. These helped CTLs clear the antigen source and die. When antigen wanes, memory precursor cells persist and form helped central (T_CM_) and effector memory (T_EM_) CD8^+^ T cells. In absence of help signals (right), antigen-specific CD8^+^ T cells undergo incomplete effector differentiation and terminally differentiated effector CTLs are lacking. Instead, predysfunctional effector CTLs are formed that are less committed (“memory-like”), i.e., have not fully unfolded their effector program and express coinhibitory receptors. In addition, formation of effector memory CD8^+^ T cells is impaired. As a result, antigen persists and continuous TCR stimulation of memory precursor cells drives their differentiation into predysfunctional CTLs that self-maintain or differentiate into terminally exhausted cells.

Illustrations in **Figures 1**–**4** were created with BioRender.

## Help Delivery During CD8^+^ T Cell Priming

Priming of CD4^+^ and CD8^+^ T cells relies on three key signals: TCR engagement by peptide/MHC complexes, costimulation by CD28 and members of the TNF receptor family, as well as specific cytokine signaling. Dendritic cells (DCs) can supply these signals, provided that the DC is of the appropriate subset and adequately activated, by pathogen- or danger-derived signals or by CD4^+^ T cells. In secondary lymphoid organs, CD4^+^ and CD8^+^ T cells engage in successive antigen-specific interactions with different DC subtypes. Migratory DCs deliver the antigen from the site of infection, while lymph node-resident DCs pick up the antigen locally. CD4^+^ and CD8^+^ T cells are initially activated independent from each other, in different regions of the lymph node by migratory conventional (c)DC1 and cDC2 subsets (12–14). After this first step of priming, a second step of priming takes place on lymph node-resident cDC1s. In this interaction, CD4^+^ T cell help is delivered that is essential for optimal differentiation of CD8^+^ T cells into CTL effector and memory cells (11) **(****Figure 1****)**. CD4^+^ and CD8^+^ T cells that have undergone the first step of priming produce specific chemokines that attract lymph node-resident cDC1 (12, 13, 15). In case the cDC1 co-presents recognizable MHC class II- and MHC class I- restricted antigens, it can relay help signals from the CD4^+^ T cell to the CD8^+^ T cell. Plasmacytoid (p)DCs likely promote this scenario by the production of type I interferon (IFN), which optimizes maturation and antigen crosspresentation by cDC1s (16).

Upon cognate contact with the CD4^+^ T cell, the lymph node-resident cDC1 gains expression of various cytokines and co-stimulatory ligands that in concert optimize the CD8^+^ T cell response (11). Interaction between CD40 ligand on the CD4^+^ T cell and CD40 on the cDC1 amplifies production of IL-12 and IL-15 by the DC, which improves clonal expansion and effector differentiation of CD8^+^ T cells (17, 18). Furthermore, CD40 signaling in DCs upregulates CD80/CD86 and CD70, which relay costimulatory signals *via* CD28 and CD27, respectively (19–21). In both CD4^+^ and CD8^+^ T cells, CD28 costimulation amplifies the TCR signal and drives cell division (22), while CD27 costimulation promotes cell survival and effector differentiation (3, 23–25). CD27 costimulation of CD8^+^ T cells is a key effector pathway of CD4^+^ T cell help. It promotes CTL differentiation and survival, likely directly, but also by increasing expression of the IL-2 receptor alpha chain, IL-2 and the IL-12 receptor, leading to autocrine IL-2 signaling and responsiveness to DC-derived IL-12 (3, 26–28). IL-21 production by CD4^+^ T cells also promotes CTL effector differentiation (29).

By transcriptomic analyses in mice, we have discovered how help signals impact effector and memory gene expression programs of CD8^+^ T cells (3, 30). At the effector stage, “helped” versus “helpless” CTLs differentially expressed about 1,000 transcripts, encoding proteins enabling critical CTL functions, such as cytotoxicity and migratory abilities. From functional studies in a tumor model, we concluded that CD4^+^ T cell help confers upon CTLs the exact properties desired for effective anti-tumor immunity, as defined by Chen and Mellman in “The cancer immunity cycle” (31). Conversely, helpless CTLs proved to have a dysfunctional phenotype characterized by low cytotoxic capacity and high expression of PD-1 and other co-inhibitory receptors (3), classifying them as “exhausted”, according to the original definition. Other authors defined by micro-array similar gene expression features in helpless CTLs, which proved to resemble exhausted CTLs, as defined in a mouse model of chronic LCMV infection (32). In conclusion, there appears to be a connection between helpless priming of CD8^+^ T cells and acquisition of the exhausted state. This connection will be clarified in this Hypothesis and Theory article.

## Antigen-Specific CD8^+^ T Cell Fates in Chronic Infection

### Exhaustion

Exhaustion of antigen-specific CD8^+^ T cells was first described in mouse models of chronic infection with LCMV (33). Exhausted virus-specific CD8^+^ T cells were defined by a diminished ability to display effector functions such as IFNγ production, and high expression of coinhibitory receptors such as PD-1. It was proposed that virus-specific effector CD8^+^ T cells gradually turn into exhausted cells upon chronic engagement of the TCR by persistent viral antigen. Observations that TCR-regulated transcription factors contribute to exhaustion led to this idea (34–36). In agreement with TCR signaling driving exhaustion, the exhausted virus-specific CD8^+^ T cell fraction was found to increase in time upon viral persistence (37). However, virus-specific CD8^+^ T cells can already show impaired effector functions from the beginning of a chronic infection, suggesting causes other than chronic antigen exposure (37). Adoptive transfer experiments demonstrated that exhausted CD8^+^ T cells in chronic LCMV infections derive from the same progenitors as memory cells and not from terminally differentiated (KLRG1^hi^) effector T cells (38). This finding suggested that exhausted CD8^+^ T cells in chronic infection do not follow a normal effector differentiation path (39).

### Predysfunction

Despite the persistence of viral antigen, not all virus-specific CD8^+^ T cells in chronic infection acquire a terminally exhausted phenotype. A subset of virus-specific CD8^+^ T cells in chronic LCMV infection was found to proliferate and give rise to terminally exhausted cells (40). Other authors defined in the same model a small “memory-like” subpopulation within the virus-specific CD8^+^ T cell pool that retained proliferative capacities and could re-expand upon secondary infection in an antigen-free host (41). Later, this proliferative population was found to express the transcription factor TCF-1 (9, 42) and the chemokine receptor CXCR5 (43, 44). These studies report that TCF-1^+^ CXCR5^+^ CD8^+^ T population is self-sustaining and constantly replenishes the exhausted CD8^+^ T cell pool. This population is described by different nomenclature (**Table 1**), but throughout this article, we will use the term “predysfunctional”. The predysfunctional population is established early in chronic infection with LCMV strain clone 13, before the peak of the T cell response, but is not seen in acute infection with LCMV strain Armstrong (51). TCF-1 is also expressed in memory T cells in acute infection, but predysfunctional TCF1^+^ T cells in chronic infection can be identified by co-expression of CXCR5, Slamf6 and PD-1 (29, 44). TCF-1 signaling represses effector differentiation and is thereby essential for generation and maintenance of predysfunctional T cells (42, 57, 59).

**Table 1:** Definitions of predysfunctional CD8^+^ T cell populations in chronic infection and cancer.

Population name	Markers	Source	References
Memory-like	TCF-1^+^	LCMV-c13	(9, 39, 41, 45)
		Human HCV	(9)
		Human melanoma	(46)
Stem-like	CXCR5^+^TIM3^−^	LCMV-c13	(44, 47)
		Human NSCLC	(48)
	PD-1^+^CD101^−^TIM3^−^	LCMV-c13	(49)
	TIM3^−^CD28^+^	Human kidney cancer	(50)
	TCF-1^+^	B16-gp33	(10)
Progenitor-like	TCF-1^high^TIM3^low^	LCMV-c13	(42)
		Human melanoma	(42)
	*Tcf7^+^Tox^+^*	LCMV-c13	(51)
Progenitor	TCF-1^+^	LCMV-c13	(52–55)
	Ly108^+^ (Slamf6^+^)	LCMV-c13	(29)
Progenitor exhausted	Slamf6^+^TIM3^−^	LCMV-c13; B16-OVA	(7)
	TCF-1^+^PD-1^+^	Human melanoma	(7)
Precursor	T-bet^high^Eomes^low^	LCMV-c13	(40)
Memory precursor-like	PD-1^−^TCF-1^+^	MC38-OVA	(56)
Precursor exhausted	KLRG1^-^PD-1^+^ Ly108^+^	LCMV-c13	(57)
	TCF-1^+^	LCMV-c13	(58)
Stem cell-like exhausted	CXCR5^+^TIM3^−^	LCMV-c13	(59)
Pre-exhausted	*GZMK^+^*, *ZNF683^+^*	Human NSCLC	(60)
Predysfunctional	multiple	Human cancers	(61)
Early dysfunctional	CD38^low^CD101^low^	ASTxCre-ER^T2^	(62, 63)
Transitional	*GZMK^+^*	Human melanoma	(64)
		Human HCC	(65)
Follicular cytotoxic	CXCR5^+^	LCMV-13	(43, 66)
		LCMV-DOCILE; HIV	(67)
		Human CHB	(68)

The listed populations have in common that they sustain the CTL response in presence of persistent antigen, and form the progenitors of the terminally exhausted population, as originally shown by Utzschneider et al. (9), Wu et al. (42), He et al. (43), and Im et al. (44) and corroborated by Miller et al. (7) and Zander et al. (29). Other cited papers consider the defined population to be predysfunctional based on the markers and the proliferative/”stem-like” phenotype described in the original papers. In the papers describing human single cell RNAseq data, the predysfunctional population is defined by intermediate expression of inhibitory receptor genes, low expression of effector-associated genes, and TCR sharing with the terminally exhausted population.

### From Predysfunction to Exhaustion

Antigenic stimulation of predysfunctional TCF-1^+^CXCR5^+^ CD8^+^ T cells can drive their differentiation into TCF-1^−^ CXCR5^−^ “terminally exhausted” cells (40, 49, 69). During this differentiation process, predysfunctional cells transiently acquire a more effector-like gene signature (49, 57, 70). Terminally exhausted CD8^+^ T cells are short-lived and display higher expression of coinhibitory receptors than TCF-1^+^ predysfunctional cells (9, 42–44). Conversion from a predysfunctional to a terminally exhausted state is associated with epigenetic and transcriptional changes involving genes encoding coinhibitory receptors, effector molecules and effector-associated transcription factors (7, 47, 70). The transcription factor TOX plays a critical role in epigenetic imprinting of dysfunction in the TCF-1^+^ subset and induces fate commitment to a terminally exhausted phenotype (51, 52, 71–73). Both the establishment of the predysfunctional population and the TOX-driven commitment to exhaustion are part of a differentiation path that is separate from effector differentiation, occurring in early stages of chronic LCMV infection (51, 57, 71). Together, these findings provide strong support for the notion that terminally exhausted T cells found in chronic infections are derived from a population of predysfunctional cells, instead of from functional effectors. Similar processes likely take place in human, where virus-specific predysfunctional and terminally exhausted CD8^+^ T cell populations have been identified in patients with chronic hepatitis C virus (HCV) infection (9). Also, CXCR5^+^ CD8^+^ T cells were found in patients infected with human immunodeficiency virus (HIV) or chronic hepatitis B virus (HBV) (67, 68).

### Reinvigoration

Importantly, PD-1 blockade unleashes the expansion potential of predysfunctional, but not terminally exhausted virus-specific CD8^+^ T cells (9, 43, 44). Predysfunctional TCF-1^+^ CD8^+^ T cells express PD-1 that supports the maintenance of this population early during chronic LCMV infection (57). Chronic virus infections (LCMV clone 13, HIV) induce chromatin accessibility and permanent demethylation of the *Pdcd1* locus (encoding PD-1), causing exhausted CD8^+^ T cells to stably express PD-1 at high levels (74, 75). Terminally exhausted CD8^+^ T cells express higher levels of PD-1 and other coinhibitory receptors than predysfunctional cells (9, 42, 43). In the terminally exhausted population, efficacy and durability of virus-specific CD8^+^ T cell reinvigoration by PD-1 blockade proved to be limited by the epigenetic landscape, including chromatin accessibility and *de novo* DNA methylation (76, 77). Taken together, these results argue that the predysfunctional virus-specific CD8^+^ T cell population in chronic infection is reinvigorated by PD-1 blockade. Predysfunctional cells respond to PD-(L)1 blockade by undergoing proliferation, as well as differentiation toward a terminally exhausted phenotype (7). During this differentiation, cells pass through an intermediate or “transitory” state, characterized by a transcriptional signature that resembles that of effector CTLs (49, 70). While these effector-like CD8^+^ T cells that are reinvigorated by PD-1 blockade are able to produce cytokines and contribute to virus control, they retain expression of inhibitory receptors and eventually convert to a terminally exhausted state upon persistent antigen exposure (49).

## Proposition: Helpless Priming Generates Predysfunctional CD8^+^ T Cells

Establishing a chronic infection in mouse models is often aided by depleting CD4^+^ T cells (33, 37, 44, 77, 78), suggesting a link between the absence of CD4^+^ T cell help and infections persisting chronically. Decreased antigen presentation and decreased costimulatory signaling by DCs during priming promote the formation of TCF-1^+^ cells, suggesting that this population may be generated as a result of suboptimal priming (45). Importantly, CD4^+^ T cell depletion in chronic LCMV infection impaired the generation of terminally differentiated effector CD8^+^ T cells, but not of predysfunctional TCF-1^+^ CD8^+^ T cells (53). This finding indicates that the predysfunctional TCF-1^+^ CD8^+^ T cell population is formed independently of CD4^+^ T cell help. We propose that this population is formed as a result of helpless priming and provide supporting evidence in this article.

As a model to study CD4^+^ T cell help for the CTL response, our group made use of a therapeutic DNA vaccination scheme in mice. We used a comparative setting with two vaccines that encode an immunodominant MHC-I restricted peptide from the human papillomavirus (HPV) E7 protein to prime CD8^+^ T cells, either with or without HPV-unrelated immunodominant MHC-II restricted peptides to induce CD4^+^ T cell help (79). Genome-wide mRNA deep sequencing of HPV-E7-specific CD8^+^ T cells at the effector stage of the CTL response yielded “Help” and “No Help” signatures (3). Helpless CTLs expressed many genes characteristic of the predysfunctional CD8^+^ T cell subset at a higher level than helped CTLs, including *Tcf7* (encoding TCF-1), *Tox*, *Pdcd1*, *Cxcr5*, and *Slamf6* **(****Figure 2A****)** (3). We therefore hypothesized that predysfunctional CD8^+^ T cells found in chronic LCMV infection are cells that have not experienced CD4^+^ T cell help during priming. To test this, we determined how predysfunctional CD8^+^ T cells defined in literature and helpless CD8^+^ T cells defined in our study are related at the gene expression level, by Gene Set Enrichment Analysis (GSEA). A published gene expression signature characteristic for the predysfunctional TCF-1^+^ CD8^+^ T cell population in chronic LCMV infection (9) in mice thus proved to be enriched in the No Help gene expression signature of antigen-specific CD8^+^ T cells from our vaccination study **(****Figure 2B****)**. Additionally, using another published dataset from chronic LCMV infection (7), we determined a “No Help score” as a measure of correlation with our No Help gene expression signature. This analysis demonstrated that predysfunctional TCF-1^+^ CD8^+^ T cells display a higher No Help score than TCF-1^−^ terminally exhausted cells, indicating that predysfunctional CD8^+^ T cells are transcriptionally more similar to helpless CD8^+^ T cells **(****Figure 2C****)**.

## CD8^+^ T Cell Dysfunction In Cancer

### The Parallel

In cancer, tumor antigen-specific CD8^+^ T cells may be chronically stimulated within the tumor micro-environment (TME), which theoretically can lead to exhaustion, as it does in mouse models of chronic virus infection. However, in the LCMV models, infection is systemic and analysis is generally focused on CD8^+^ T cells from the spleen. This milieu is distinct from the TME in partially undefined aspects. In both environments, specific conditions are created by interplay between infected cells or growing tumor cells, immune cells and non-immune cells. Intratumoral CD8^+^ T cells are known to be exposed to various suppressive immune cell types, inhibitory molecules, hypoxia, metabolites and nutrient deprivation (2).

### Mouse Models

Using a mouse model of tamoxifen-inducible liver cancer, it was shown that tumor antigen-specific CD8^+^ T cells taken from the TME early during tumorigenesis could be reinvigorated by PD-1 blockade or recall in an antigen-free host. Late in tumor development, however, these cells could no longer be rendered functional. It was found that tumor-specific CD8^+^ T cells in the TME over time acquire a fixed dysfunctional phenotype (62). Follow-up research in this model showed that tumor-specific CD8^+^ T cells in the TME first attain a reversible dysfunctional state and next enter a epigenetically fixed dysfunctional state (63). These data are in agreement with a transition from predysfunction to exhaustion.

In a murine melanoma model, single-cell transcriptomics revealed that among CD8^+^ tumor infiltrating lymphocytes (TILs), TCF-1^+^ predysfunctional and TCF-1^−^ terminally exhausted cell subsets can be discerned that are analogous to those defined in chronic LCMV infection. Adoptive transfer experiments demonstrated that TCF-1^+^ CD8^+^ T cells can persist long-term inside a tumor and give rise to terminally exhausted cells (7). Like in chronic infection, transcriptional and epigenetic changes underlying this conversion depended on the transcription factor TOX (72, 73).

### Human Cancer

Also in human cancer, there is increasing evidence for the existence of predysfunctional and terminally exhausted CD8^+^ T cell populations. In non-small cell lung cancer (NSCLC), CXCR5 expression was selectively found on CD8^+^ TILs and not on CD8^+^ T cells from healthy tissue or blood (48). In kidney cancer, TCF-1^+^TIM3^−^CD28^+^ predysfunctional TILs were found to reside in niches that are rich in antigen-presenting cells, while PD-1^+^TIM3^+^ terminally exhausted cells were distributed throughout the tumor tissue. Transcriptional and epigenetic profiles of these human TIL subsets proved to be similar to those described in the mouse. Importantly, TCR repertoire overlap between the two populations indicated that TCF-1^+^ predysfunctional TILs are indeed the progenitors of terminally exhausted TILs (50). TCR repertoire overlap between a terminally exhausted TIL population, characterized by high expression of coinhibitory receptor genes, and a predysfunctional TIL population, characterized by expression of *GZMK*, was also found in human melanoma (64), NSCLC (60), colorectal cancer (CRC) (80) and hepatocellular carcinoma (HCC) (65). These findings are consistent with a model where also in human cancer, exhausted TILs derive from a predysfunctional population. However, a strict division of the human TIL pool into predysfunctional or terminally exhausted may be an oversimplification. Rather, CTL dysfunction in human TILs covers a spectrum of differentiation states, ranging from predysfunctional to terminally exhausted (61).

The question remains whether the active CTLs that display effector functions in human tumors are generated from a separate CD8^+^ T cell pool, or are connected to the (pre)dysfunctional pool. In CRC, HCC and NSCLC studies, TCR sharing was found between *GZMK^+^* predysfunctional TILs and *CX3CR1^+^* effector populations from blood and normal tissue (60, 65, 80). These results support a model in which the predysfunctional population forms a branchpoint from which differentiation trajectories of effector versus exhausted CD8^+^ T cells emanate, possibly reflecting CD8^+^ T cells after the first step of priming that subsequently receive CD4^+^ T cell help, versus CD8^+^ T cells that do not. However, it was not determined in those studies whether the T cells that shared TCRs were tumor-specific. In melanoma, intratumoral *GZMH^+^* effector CTLs did not share TCRs with the predysfunctional or exhausted CD8^+^ TIL population, indicating that they formed a separate lineage (64). Interestingly, in this study, tumor reactivity was enriched in the dysfunctional but not in the cytotoxic TIL population, suggesting that the cytotoxic population consists of bystander cells that do not recognize the tumor, as was demonstrated before (81, 82). These data argue that in melanoma, persistent tumor antigen recognition drives the conversion of helpless tumor-specific TILs from the predysfunctional to the terminally exhausted state, while the tumor may also harbor helped bystander cells with an effector phenotype (61). Whether tumor-specific dysfunctional TILs can differentiate within the TME into competent effector CTLs remains to be investigated.

### Reinvigoration

In mouse models of melanoma, the TCF-1^+^ predysfunctional CD8^+^ TILs proved to be the responders to PD-1 blockade and necessary for tumor control (7, 10, 56). In melanoma patients, an increased fraction of TCF-1^+^ predysfunctional CD8^+^ TILs is a positive predictor for response to PD-(L)1 targeted therapy (7, 46). In a murine liver cancer model, CD101 and CD38 marked predysfunctional versus terminally exhausted TILs. These markers were heterogeneously expressed by PD-1^high^ TILs from melanoma and NSCLC patients, suggesting that the human PD-1^high^ TIL population consists of a mixture of predysfunctional and terminally exhausted cells (63).

## Helplessness and Predysfunction in Cancer

CD4^+^ T cell help is less likely to be delivered in cancer than in infection for the following reasons: Tumor cells generally do not express PAMPs and may only exude DAMPs under specific circumstances. Therefore, they are less likely to activate migratory DCs than infected cells. Furthermore, in the suppressive TME, migratory cDC2s, which are essential for the priming of CD4^+^ T cells (83), are reportedly suppressed by Tregs, resulting in suboptimal priming of CD4^+^ helper T cells in the tumor-draining lymph node (84). Also, DC-activating signals such as type I IFN that promote crosspresentation functions of the lymph node-resident cDC1 (16), are often lacking. In the blood of melanoma patients, tumor reactivity of CTLs was found to be enriched in the PD-1^+^ population (85). These data led us to hypothesize that helpless priming may contribute to the dysfunctional phenotype of CD8^+^ T cells in cancer.

To test this hypothesis, we performed bioinformatic analyses using our previously defined No Help versus Help signatures of mouse CD8^+^ T cells and datasets from mouse and human cancer. GSEA showed that gene sets characteristic of predysfunctional TCF-1^+^ CD8^+^ TILs from a gp33 antigen bearing B16 melanoma mouse model (10) were enriched in the No Help gene expression signature **(****Figure 3A****)**. In an ovalbumin (OVA) antigen-bearing B16 melanoma model from a different research group (7), TCF-1^+^ CD8^+^ TILs displayed a higher No Help score than TCF-1^−^ CD8^+^ TILs **(****Figure 3B****)**. These results indicate that also in mouse cancer models, dysfunctional TCF-1^+^ CD8^+^ TILs display a gene expression profile that resembles that of helpless cells. In NSCLC patients, the presence of PD-1^high^ TILs was a positive predictor of response to PD-1 blockade therapy. Importantly, PD-1^high^ TIL displayed higher intrinsic tumor reactivity compared to TIL populations with intermediate or no PD-1 expression from the same tumor (8). We used the published gene expression profiles from these matched TIL subsets to calculate their No Help score. Among these patients’ TIL populations, the transcriptome of PD-1^high^ TILs was most similar to that of helpless vaccine antigen-specific CD8^+^ T cells **(****Figure 3C****)**. These data from human cancer support our hypothesis that dysfunctional tumor-reactive CD8^+^ T cells are cells that have lacked help during priming.

## Helpless Dysfunction Model

We present a novel model posing that virus-specific or tumor-specific, predysfunctional TCF-1^+^ CD8^+^ T cells in chronic infection or cancer result from priming in the absence of CD4^+^ T cell help. CD4^+^ T cell help delivered during priming optimizes effector differentiation of antigen-specific CD8^+^ T cells (3, 53). Additionally, CD4^+^ T cell help promotes effector memory CD8^+^ T cell (T_EM_) generation, and renders these T_EM_ cells more effector-like on a per-cell basis (30). These results are in line with a previously proposed progressive differentiation model for primed CD8^+^ T cells (86), adding that CD4^+^ T cell help shifts differentiation of primed CD8^+^ T cells toward a more effector-like state **(****Figure 4****)**.

By optimizing CTL function, CD4^+^ T cell help contributes to antigen clearance, which is necessary for proper memory formation (87, 88). CD4^+^ T cell help also promotes the long-term maintenance of T_CM_ cells and is necessary for open configuration of gene loci encoding CTL effector molecules in memory CD8^+^ T cells (30, 89, 90). The epigenetic imprinting induced by help signals during priming allows memory cells to rapidly exert effector functions upon reactivation in a CD4^+^ T helper cell-independent manner (30, 91).

In the absence of CD4^+^ T cell help, effector differentiation of CD8^+^ T cells is incomplete, resulting in predysfunctional CTLs that have limited cytotoxic and migratory potential and express coinhibitory receptors (3, 32), which prohibits antigen clearance. The chronic stimulation of memory precursor cells impairs the formation of a memory pool and instead drives their differentiation into predysfunctional CTLs, as seen in chronic infection and cancer (39, 54). These predysfunctional TCF-1^+^ cells have self-maintaining properties and form the progenitors of the terminally exhausted TCF-1^−^ CD8^+^ T cell pool (58). Exhausted CD8^+^ T cells differ in their epigenetic and transcriptional states from predysfunctional CD8^+^ T cells. They have a further developed effector differentiation program, but are fixed in their dysfunctional state (55).

## Overcoming CTL Dysfunction By Help Signals

Based on our model, we propose that in chronic infection and cancer, CTL dysfunction can be overcome by help signals. In that scenario, help signals would enable the CTLs to progress further toward a terminal effector differentiation state. Adoptive transfer of CD4^+^ T cells has been shown to increase proliferation of pre-existing TCF-1^+^ CD8^+^ T cells in chronic LCMV infection (53). Also, adoptive transfer of IL-21-producing CD4^+^ T cells into tumor-bearing mice induced generation of a CX3CR1^+^ effector CD8^+^ T cell pool, leading to improved tumor control (29). Using help signals to alleviate CTL dysfunction is not yet incorporated into clinical protocols. In the clinic, PD-1 blockade is used as method to “reinvigorate” dysfunctional CTLs.

We here propose that PD-1 blockade recapitulates aspects of CD4^+^ T cell help and acts on the predysfunctional/helpless CD8^+^ T cell population. As reviewed in the preceding sections, in chronic LCMV infection and cancer, PD-1 blockade induced proliferation of predysfunctional TCF-1^+^ CD8^+^ T cells. The question is whether PD-1 blockade is sufficient to overcome lack of help and—by association—to convert predysfunctional CTLs into fully functional effectors. In chronic LCMV infection, established through transient CD4^+^ T cell depletion, PD-L1 blockade promoted differentiation of predysfunctional CD8^+^ T cells into transitional cells that displayed a more effector-like phenotype and contributed to virus control. However, eventually these cells became terminally exhausted (49). Blockade of the PD-L1/PD-1 axis in a helpless setting increases the magnitude of the antigen-specific CD8^+^ T cell response, but in contrast to CD4^+^ T cell help, it did not rescue the formation of the effector population that conferred protection against chronic infection and cancer (29). These results suggest that predysfunctional/helpless cells cannot be rescued by PD-1 blockade alone.

The prevailing view is that PD-1 blockade relieves pre-existing dysfunctional CTLs from suppression in the TME. However, accumulating data argue that PD-1 blockade can also facilitate *de novo* CTL priming. Firstly, PD-(L)1 targeted immunotherapy can be effective while PD-L1 is not expressed in the tumor (92). Secondly, PD-1 signaling impedes TCR as well as CD28 signaling, indicating that it can also impact on costimulation at the T cell/DC interface (93). In agreement with this, tumor regression upon PD-1 blockade in mouse colon carcinoma depended on CD28 co-stimulation (94). Thirdly, the response to PD-1 blockade in mouse colon carcinoma was found to depend on influx of newly activated CD8^+^ T cells from tumor draining lymph nodes (95).

Recent data from human cancer also argue that PD-1 blockade promotes CD8^+^ T cell priming: In basal cell carcinoma, new CD8^+^ T cell clones entered the upon tumor PD-1 blockade (96). TCR repertoire analysis argued that these clones pre-existed in blood and entered the tumor after treatment (97). PD-1 is expressed rapidly after stimulation of naive CD8^+^ T cells, and inhibits effector differentiation during priming (98). We found that in the CD4^+^ T cell help-dependent second step of priming, CD8^+^ T cells downregulate PD-1, whereas helpless cells maintain PD-1 expression (3). This supports a model in which PD-1 serves as a checkpoint in the two-step T cell priming process.

We have shown in the mouse vaccination model, that the effects of CD4^+^ T cell help on the CTL response could be mimicked by combined PD-1-blockade and CD27 agonism (99). We and others have shown that delivery of CD4^+^ T cell help is highly dependent on CD70-CD27 signaling and CD27 agonism installs a large part of the Help gene signature into CD8^+^ T cells during priming (3, 20, 24, 25). The combined effect of PD-1 blockade and CD27 agonism likely recapitulates combined CD28 and CD27 costimulation that are known to complement each other in generation of the CTL effector pool (23). The collective data make a strong case for combining CD27 agonism with PD-(L)1 blockade in cancer immunotherapy.

## Concluding Remarks

We here present our hypothesis that CD8^+^ T cell priming in the absence of CD4^+^ T cell help leads to CD8^+^ T cell dysfunction. We pose that exhausted antigen-specific CD8^+^ T cells observed in infection and cancer derive not from previously active CTLs, but from helpless CD8^+^ T cells that emerge from the priming process in a dysfunctional state. We pose that provision of CD4^+^ T cell help, or the key signals that recapitulate help for CD8^+^ T cells will be crucial for the development of effective immunotherapeutic strategies in chronic infection and cancer. In immunotherapy, reverting exhausted cells back to a functional phenotype is considered an important challenge (1). Alternatively, we argue that in patients with immunogenic cancer types, *de novo* priming of helped CD8^+^ T cells will be beneficial for tumor control. For this purpose, potential approaches are antigen-agnostic PD-1/CD27 targeting or antigen-informed therapeutic vaccination. Such vaccines should contain MHC-I and MHC-II epitopes to activate both CD8^+^ and CD4^+^ T cells. Other strategies include specific targeting of antigens and activation signals to XCR1^+^ cDC1s. In these approaches, evaluation of the transcriptional help signature in tumor-specific CD8^+^ T cells is a potential diagnostic tool.

## Data Availability Statement

Publicly available datasets were analyzed in this study. This data can be found here: https://www.ncbi.nlm.nih.gov/geo/.

## Author Contributions

JuB performed data analysis and wrote the paper. ST performed data analysis. JaB contributed to writing the paper. HE critically reviewed the paper. All authors contributed to the article and approved the submitted version.

## Funding

This work was financially supported by grant 11079 from the Dutch Cancer Society.

## Conflict of Interest

HE and ST were employees of Aduro Biotech. HE has stocks and/or stock options in Aduro Biotech, Inc.

The remaining authors declare that the research was conducted in the absence of any commercial or financial relationships that could be construed as a potential conflict of interest.
